# Rhino-Plasty

**Published:** 1890-05

**Authors:** Geo Goodhue

**Affiliations:** Dayton, Ohio


### Rhino-Plasty.

BY DR. GEO. GOODHUE, DAYTON, OHIO.

Of all parts of the face that give character and expression, the nose ranks pre-eminently first; and hence its loss or mutilation results in the greatest possible deformity. In the olden time, when it was the custom to punish evil doers by cutting off the nose, to the deformity was added the stigma of crime.

At the present time in civilized countries the loss of this organ is so generally due to syphilis, that now to this deformity is added the brand of immorality. Hence, it is not surprising to find that from the earliest time down to the present, restoration of this organ has been demanded of surgeons by operative interference. The art has been practiced in India from time immemorial. Celsus speaks of the operation in his cyclopædia of medicine. In the sixteenth century a new method of operation was introduced by Taliacotius, an Italian surgeon, who wrote a work on nasal auto-plasty, which is still in good repute. In the present century it has been largely practiced in Europe and with marked success. In America, too, it has had many enthusiastic advocates, and been practiced with brilliant results, especially in the hands of Dr. Warren, of Boston, and Prof. Pancoast, of Philadelphia.

In rhino-plastic operations there are two methods from which we may choose, the Indian and the Italian. In the Indian operation the tissue is taken from some contiguous portion of the face; in the Italian, from some distant portion of the body, generally the arm. The so-called French method is but a modification of the Indian, and the German a modification of the Italian, neither deserving the honor of a distinct name.

When crime was punished by cutting off the nose, the portion removed was often replaced and so frequently found to grow again that the excised portion was ordered to be burned.

But attempts to transplant foreign tissue upon nose stumps refreshened, without leaving an arterial supply, has proved futile, and whatever method we apply one end of the flap is left attached until the other end has united with the nose. The

Italian method, on account of the painful confinement of the head and arm, is now seldom employed, and we choose the older or Indian method, with perhaps modifiations, as was the case in the operation which I will now report.

The patient, female, was 46 years old, of German descent, married, and mother of one child, and in quite feeble health.

She had several large scars on one arm and one on her left cheek; and end of nose, including both alæ and cartilages, gone. She was sent to me in the early part of June, 1887, by Dr. Hooven, to get my opinion in regard to operation for restoring the lost member. In conferring with the physicians under whose treatment she had been for the last three years, I found that these sores were of a specific character and had been very obstinate in healing, especially the one on the end of the nose: but finally they all yielded and she was left with this terrible deformity.

Although she was unconscious of the cause of her trouble and not especially æsthetic in her tastes, her appearance was so wonderfully marred that she could not tolerate the gaze of passers-by which her deformity attracted, and fairly besought me to make an attempt to build her a new nose.

I reluctantly complied with her request, knowing that several cases had resulted fatally, and many attempts had fallen short of the end desired, and the patient was left the original deformity plus a scar of greater or less magnitude on cheek or forehead from which the material was borrowed for construction of a new nose. Having decided to make the attempt, my patient was put on iodide of potash for eight weeks, which very materially improved her general condition.

On August 18, 1887, with the valuable assistance of Drs. J. M. Weaver and H. O. Collins, I made my first operation. I chose the right cheek as the site from which to obtain tissue for transplantation because of the presence of scars on the left cheek, and there would not be the necessity of twisting the pedicle of flap as would be the case had I selected the forehead; and, hence, I would be more certain of abundant arterial supply, which, of course, is of the utmost importance in the transplanting of so large a flap as this operation necessitates.

I had previously made a leather flap of proper dimensions to fit the nose stump and restore as perfectly as possible the size and form of the lost organ. This leather flap, when increased about one-fourth to allow for tissue contraction, measured four inches in length and two and three-quarter inches at its greatest breadth. This model was placed on the cheek and outlined by nicks with a scalpel which served as a guide in cutting my flap for transplantation, which included all the tissues overlying the muscles; care being taken not to wound the angular artery.

As soon as the hemorrhage had largely ceased and wound had been bathed with a 1 to 5,000 solution of bichloride of mercury, the flap was returned to its original site and covered with cotton soaked in a hot bichloride solution of the same strength, in order that the flap might not become chilled. The nose having been plugged with cotton, its edges were pared for the reception of the new material, and at this point we met with the most tedious part of the whole operation. The hemorrhage, while it was not profuse, was constant and seemed almost interminable.

To insure primary union, torsion and bruising of the bleeding surfaces was carefully avoided, and hot water and gentle pressure were the only hæmostatic agents that we felt justified in employing.

Finally the new tissue was firmly and accurately stitched to the nose stump by ten sutures of antiseptic silk; rubber tubes were introduced to maintain the natural calibre of the nostrils. Iodoform was plentifully applied covered by antiseptic gauze and all overlaid with an abundance of borated cotton held in place by adhesive plaster.

My patient rallied well from the anæsthetic, although the hemorrhage had necessitated its employment for nearly three hours. She suffered no pain and very little inconvenience. On the fourth day dressings were removed and primary union found to have taken place everywhere except at the end of the columna nasi, which was remedied by subsequent operation. Five of the sutures were removed and dressings reapplied. On the sixth day dressings were removed and also the remaining sutures. Nothing more was done for four weeks, when the pedicle was severed and a portion of it carried back to its original position to take the place of the cicatricial tissue at angle of nose, which was removed to make room for it.

After several days had elapsed and it had been demonstrated that the new material was receiving ample nutrition for its support from the nose itself, its upper edge was pared and carefully adapted to the upper portion of the nose stump which had necessarily been previously overlapped by the whole thickness of the cheek flap. This was stitched with silk and dressed under the most careful antiseptic precautions. In some way my patient at this time contracted a severe cold, had high fever and was greatly prostrated for three or four days. On the fourth day dressings were removed and primary union had taken place at the central and most important portion; but on each side, for a short distance, it had failed to do so. These small wounds healed, however, very quickly by granulation, with scars invisible except by close scrutiny. Several minor operations were done subsequently, in way of ornamentation, and we had a nose, which, while not as comely as nature's own, was still a fair substitute for the original. My patient was ordered to take iodide of potash occasionally during the remainder of her lifetime. No tendency to a recurrence of the old trouble has appeared since the operation, now over two years. —*Exchange.*

WOMEN DOCTORS IN GERMANY.—An association of German ladies, at a meeting a short time ago, passed a resolution agreeing to a petition being presented to the divers German governments praying for permission to be granted to women to study medicine. The petition so far has been flatly refused by Prussia, Wurtemburg, Saxony, the Duchies of Hesse-Darmstadt and Saxe-Weimar. This is not surprising in view of the hyper-congested condition of the medical profession in the fatherland. The States may be expected to know what is best for themselves.—*Med. Press and Circular*, Jan. 15, 1890.

